# Development and evaluation of the evidence-based medicine program in surgery: a spiral approach

**DOI:** 10.3402/meo.v19.24269

**Published:** 2014-04-25

**Authors:** Melih Elçin, Sevgi Turan, Orhan Odabaşı, İskender Sayek

**Affiliations:** 1 Department of Medical Education and Informatics, Faculty of Medicine, Hacettepe University, Ankara, Turkey; 2 Department of Surgery, Faculty of Medicine, Hacettepe University, Ankara, Turkey

**Keywords:** evidence-based medicine, spiral curriculum, medical education

## Abstract

**Background:**

Evidence-based medicine (EBM) aims to provide skills that help physicians answer clinically important questions, determine new evidence, and incorporate the acquired knowledge in practice. EBM skills are necessary for the practice of modern medicine, since physicians should use up-to-date knowledge and information to justify their medical decisions.

**Purpose:**

We aimed to evaluate the EBM program implemented at Hacettepe University School of Medicine.

**Methods:**

In 2004, a spiral program for the teaching and practice of EBM was developed for the first 3 years of medical school. Following this program, a practice of EBM was included in the fourth year during the surgery clerkship, after an introductory lecture. The students worked within collaborative teams of 3–5 and practiced EBM with actual cases seen in the surgical service in which they were involved. Each student was asked to complete a questionnaire that evaluated the more theoretical program from the first 3 years and the practical application in the fourth year.

**Results:**

Nearly half of the students stated that the preclinical years of the EBM program were ‘adequate’, but only 30% of the students indicated that the program was practical. They stated that ‘more practical approaches were used in the fourth year, whereas more theory-based approaches were used during the preclinical years’. More than 75% of the students declared that the practice of EBM in the fourth year was useful and appropriate for team-based learning.

**Conclusions:**

The EBM program was evaluated as ‘adequate’. EBM courses should be included in the entire curriculum in an integrated manner. The students understand the main philosophy of EBM in the clinical year when involved in its practical application with actual patients.

Evidence-based medicine (EBM) is an important part of decision making and requires an understanding and use of scientific principles and methodologies (1). EBM helps physicians to integrate their experience and individual expertise with the best evidence, integrating research and clinical practice (2). Asking appropriate questions, acquiring appropriate evidence, appraising the evidence, and applying the information to patient care are the major components of EBM (3). These skills are necessary and of utmost significance for the practice of modern medicine because physicians should be aware of the latest developments and use up-to-date knowledge and information to justify their medical decisions (4).

In general, there is a consensus on incorporating EBM principles throughout the undergraduate medical education (4, 5). In response to this recommendation, the teaching of EBM has become widespread in medical education at the undergraduate and graduate levels (6) with a variety of methods and content (7). Studies have aimed to evaluate the impact of EBM programs on the students’ knowledge level (8–11), performance in searching skills (12, 13), EBM skills (6, 9–11, 13, 14), attitudes toward learning and applying EBM (10, 11, 13, 14), and self-efficacy in using EBM (15).

Although a variety of curricula have been developed to teach EBM, it was reported that most of them took place in classroom settings that feature teacher-centered didactic activities, while few curricula have extended EBM instruction longitudinally throughout the clinical rotations (15, 16). A recent systematic review assessing methods of teaching the EBM process to surgical residents showed that most of the studies used structured lectures and some types of journal clubs (17). However, it was asserted that when EBM was taught in this manner, the teaching appeared not to influence medical practice and patient care (15).

It is widely accepted that essential EBM skills should be taught early in the undergraduate medical curriculum (6, 15, 18). When the EBM skills were presented during the preclinical years of the medical curriculum, students successfully transferred the knowledge (7, 11, 19). However, it is not known whether students can independently apply these skills in the clinical setting (7, 19). In fact, preclinical students have limited opportunities to apply EBM to patient care (15). Implementing an EBM program in a clinical clerkship may provide students an opportunity to practice these skills on actual patients (6). There is also evidence that an EBM curriculum combining an initial short course and integration of EBM practice with clinical activities resulted in sustained increases in perceived and measured EBM knowledge (16). Thus, it is recommended that the teaching and learning of EBM is moved from the classrooms to clinical settings (7, 20). This is possible when a spiral approach is used in designing the curriculum. In a spiral curriculum, there is iterative revisiting of topics throughout the course, and topics are revisited at numerous levels of difficulty. Thus, students can relate new learning with previous learning, and the competence of students increases with each visit to a topic (21).

With the above in mind, we designed a program based on the spiral approach to develop EBM skills throughout the undergraduate medical education. The EBM program was initiated the first year and continued for the 3 years of medical school, and was then integrated with clinical experience during the surgery clerkship on actual patients with whom the students were involved. We used Khan and Coomarasamy (20) hierarchy classification to describe our EBM program. They developed the hierarchy for teaching and learning of EBM in terms of its educational effectiveness. They classified three levels as: Level 1, interactive and clinically integrated activities; Level 2(a), interactive but classroom-based activities; Level 2(b), didactic but clinically integrated activities; and Level 3, didactic, classroom or standalone teaching. They concluded that interactive and clinically integrated teaching and learning activities (Level 1) provide the basis for the best educational practice in this field. According to Khan and Coomarasamy's (20) classification, our program is Level 1, which is defined as an interactive and clinically integrated activity in clinical years, and Level 2(a), indicating interactive but classroom-based activities in the preclinical years. In this study, we aimed to evaluate the EBM program based on the students’ perceptions after the completion of the program.

## Methods

### The setting and program

#### Curriculum model in Hacettepe University Faculty of Medicine (HUFM)

The medical education in Hacettepe University lasts 6 years, with the first 3 years as the preclinical phase and the last 3 years as the clinical phase. A system-based curriculum is used in Hacettepe University. The curriculum is performed as subject committees based on body and organ systems in Phases 1, 2 and 3 and clinical clerkships in Phases 4, 5 and 6.

#### Evidence-based medicine program

We developed an EBM program based on a spiral approach that is continued throughout the first 3 years of medical school and then integrated with clinical experience during the surgery clerkship. The EBM program is described in detail below.

#### Preclinical years EBM program

The EBM program has been carried out in the preclinical years (first 3 years) at HUFM since 2004. This introductory course included 8 student contact hours each year. The preclinical EBM program was aimed to achieve competency in information seeking by using an electronic database in the first year; knowledge and familiarity with basic concepts of EBM such as defining EBM, formulating a well-built search question based on a standardized case scenario, and identifying and reviewing EBM search strategies and resources in the second year; and critical reading and appraisal of the literature in the third year. Students studied EBM within a small group. As the preclinical years do not include any patient contact, the program was implemented with the discussion of paper-cases in small groups in the second and third years. The steps of the preclinical EBM program were:

1. First year: A lecture and practicum of information seeking (8 hours).
2. Second year: A lecture and homework for practice of EBM principles with paper-cases with collaborative teams (8 hours).
3. Third year: A discussion session for reading and discussing different types of studies (a randomized control study, a systematic review and a case study) (8 hours).

#### Clinical year EBM program

Students take the clinical EBM program in groups of approximately 20 students each (range: 17–20), divided into collaborative teams of 3–4 students under the supervision of a faculty member (IS). The clinical EBM program takes 10 student contact hours, and it includes both theory and practice. It was designed to develop students’ critical appraisal skills with an emphasis on diagnosis and treatment. The difference of the clinical year program from the preclinical year program was the study on actual patients with whom the students were involved. During the course, students generated a clinical question, searched for evidence in the literature addressing that question, and critically appraised the articles. Under the supervision of a faculty member (IS), each group presented their results to the larger group and discussed the evidence and conclusions they reached.

The steps of the clinical EBM program were:

1. A brief introductory lecture for recalling the principles of EBM.
2. Practicum of EBM with clinical cases selected in surgery with collaborative teams, in which students:
  - Formulate a clinical question
  - Search the evidence
  - Critically appraise the evidence
  - Integrate the evidence into practice
  - Evaluate the evidence
3. Report and presentation of the results of the team study in relation to the patient they are involved with on the floor.

### Subjects

The study was conducted with fourth year students who attended the general surgery clerkship at HUFM. Ninety-six students who attended the clinical EBM program participated in the study.

### Instrument

In order to evaluate the EBM program, students were asked to complete a voluntary and anonymous questionnaire at the end of the EBM program in surgery clerkship. The questionnaire included 12 closed questions on the preclinical and clinical EBM program. Students were also asked their general opinions and suggestions with respect to the preclinical and clinical EBM program in two open-ended questions.

### Analysis

The number and percentage of the data were calculated. In addition, we decoded open-ended questions to identify themes related to the students’ opinions and suggestions on the preclinical and clinical EBM program.

## Results

The content of the EBM course during the preclinical years included the importance of accessing information, the concepts of information literacy and information literate, the criteria for assessing reference books/journals/articles/web pages, search engines, health databases, initials of web pages, and cues for searching information. Half of the students indicated that they recalled the ‘importance of accessing information’ and ‘search engines’. They attested that they mostly or somewhat recalled ‘health database’ and ‘cues for searching information’. However, a remarkable number of students indicated that they did not recall the criteria for assessing reference books (18.8%), journals (34.4%) and web pages (26.0%) (Table 1).

**Table 1:** Students’ views about retention of content of evidence-based medicine course in the preclinical years

	I recall					
	
	Never		Somewhat		A great deal	
	
EBM course in the preclinical years	n	%	n	%	n	%
Importance of accessing information	–	–	45	46.9	51	53.1
Information literacy	13	13.5	55	57.3	28	29.2
Information literate	16	16.7	57	59.4	23	24.0
Criteria for assessing reference books	18	18.8	64	66.7	14	14.6
Criteria for assessing reference journals	33	34.4	46	47.9	17	17.7
Criteria for assessing articles	8	8.3	56	58.3	32	33.3
Search engine	4	4.2	35	36.5	57	59.4
Health database	5	5.2	46	47.9	45	46.9
Initials of web pages	13	13.5	68	70.8	15	15.6
Criteria for assessing web pages	25	26.0	59	61.5	12	12.5
Cues for searching information	3	3.1	52	54.2	41	42.7

Half of the students indicated that the content regarding ‘importance of accessing information’ and ‘search engines’ was sufficient. However, a remarkable number of students indicated that the content about the criteria for assessing reference books (35.4%), journals (44.8%) and web pages (35.4%), and initials of web pages (41.7%) was insufficient (Table 2). Students prepared assignments for the application of EBM principles with a paper-case in the second year. Half of the students stated that practice of EBM with the assignment was moderately sufficient.

**Table 2:** Students’ views on the adequacy of the evidence-based medicine course in the preclinical and clinical years (in surgery clerkship)

	Insufficient		Moderately sufficient		Sufficient	
	
	n	%	n	%	n	%
EBM course in the preclinical years						
Importance of accessing information	3	3.1	42	43.8	51	53.1
Information literacy	15	15.6	58	60.4	23	24.0
Information literate	20	20.8	57	59.4	19	19.8
Criteria for assessing reference books	34	35.4	40	41.7	22	22.9
Criteria for assessing reference journals	43	44.8	33	34.4	20	20.8
Criteria for assessing articles	19	19.8	40	41.7	37	38.5
Search engine	4	4.2	47	49.0	45	46.9
Health database	16	16.7	46	47.9	34	35.4
Initials of web pages	40	41.7	42	43.8	14	14.6
Criteria for assessing web pages	34	35.4	43	44.8	19	19.8
Cues for searching information	14	14.6	54	56.3	28	29.2
Practice of EBM with homework	18	18.8	50	52.1	28	29.2
EBM course in the clinical years						
Recalling prior knowledge (presentation)	4	4.2	28	29.2	64	66.7
Content of the course	1	1.0	41	42.7	54	56.3
Cases for EBM practice	7	7.3	30	31.3	59	61.5
Practice of EBM	5	5.2	34	35.4	57	59.4

Students also assessed the adequacy of EBM courses during the surgery clerkship on actual patients. The majority of students stated that content regarding ‘recalling prior knowledge’ (66.7%), ‘content of the course’ (56.3%), ‘cases for the EBM practice’ (61.5%), and ‘practice of EBM’ (59.4%) was sufficient (Table 2). Most of the students also specified that they utilized the knowledge and information obtained in the EBM course (77.1%), and that the course was appropriate for teamwork and contributed to the achievement of teamwork skills (Table 3).

**Table 3:** Students’ views on utility and appropriateness of teamwork during the evidence-based medicine course in the clinical years (in surgery clerkship)

	n	%
Utility of evidence-based medicine course		
Never	3	3.1
Somewhat	19	19.8
A great deal	74	77.1
Appropriateness of teamwork		
Never	6	6.3
Somewhat	17	17.7
A great deal	73	76.0
Contribution to learning of teamwork skills		
Never	2	2.1
Somewhat	20	20.8
A great deal	74	77.1
Total	96	100.0

Students were asked their views on the EBM courses during the preclinical and clinical years with open-ended questions, and 48 students answered the open-ended question about evaluation of the EBM courses in the preclinical years. Students expressed their views within seven themes: practice in the EBM course (25), utility of the EBM course (8), value of the EBM course (5), tutor roles in the course (4), content of the course (3), integration of the content (2), and student's role in the course (1). In the open-ended questions, students were also asked their views of the EBM course during the surgery clerkship, and 54 students answered the questions. Students expressed their views on practice in the course (20), utility of the course (25), and integration of content (9) themes (Table 4). The students’ views are summarized below:

Half of the students explained their views in relation to practice of the EBM course during the preclinical years, and indicated that practice of EBM during the course was inadequate; however, they stated that they had at least become acquainted with reading the literature. They reported that clinical experience is essential in EBM.

Eight of the students indicated that they utilized the EBM course during their preclinical years. However, because of inadequate practice and prerequisite behavior of the students, they declared that while they had not benefited fully, they had learned how to search for evidence in the literature.

In this regard, students stated that the program in surgery clerkship was more effective. Almost half of the students stated that they benefited from the EBM course during the surgery clerkship. Further, they stated that the practice of EBM was effective and complimentary to the program during the first 3 years and that the supervisor was motivating.

Twenty of the students explained their views in relation to practice of the EBM course during the surgery clerkship. Although they stated that the practice was better than the course in the preclinical years, they requested to study more cases with better facilitation of their study. Students declared that they integrated their previous learning about EBM when they took the EBM course during the surgery clerkship.

**Table 4:** Themes of students’ views and suggestions for development of the EBM course in the preclinical and clinical years (in surgery clerkship)

	Preclinical years	Surgery clerkship
Themes of students’ views		
Students	1	–
Integration	2	9
Course content	3	–
Tutors	4	–
Value of course	5	–
Utility of course	8	25
Practice in the course	25	20
Total	48	54
Themes of students’ suggestions		
Assessment	1	–
Library	1	1
Assignments	2	–
Tutor	4	2
Timing	4	3
Methods	7	2
Practice	7	4
Extension	1	6
Total	27	17

Students were asked for their suggestions in developing the EBM course during the preclinical years and surgery clerkship. Students expressed their views within ‘extension’, ‘practice’, ‘methods’, ‘timing’, ‘tutors’, ‘assignments’, ‘library’, and ‘assessment’ themes (Table 4). Seven of the students suggested that there should be more practice in the EBM course during the preclinical years. Some students suggested methods for designing the EBM course during the preclinical years, the roles of the tutors in the preclinical years, and stated that its effectiveness was tutor- and student-dependent. They again suggested there should be more practice in the clinical setup.

## Discussion

Based on our results, it appears that an important part of the knowledge presented in the preclinical EBM course was not remembered or recalled by most of the students. Students declared that they did not recall some content of the preclinical EBM course (Table 1). The content that could be practiced later in the daily routine, such as using search engines, had a higher persistence. The assessment of the adequacy of the EBM courses in the preclinical years was similar, in that students perceived inadequacy in training of the EBM content when they did not have the opportunity for its clinical application.

Students indicated that content regarding assessing reference books, journals and web pages was insufficient during the preclinical years (Table 2). The lack of preliminary knowledge in this regard and the novelty of the content might have contributed to the lack of permanent knowledge. However, the majority of students stated that the content of the EBM course in surgery clerkship was sufficient, that the course was appropriate for collaborative teamwork, and that it contributed to achieving teamwork skills (Table 3).

Students declared that they benefited from the EBM course during the surgery clerkship. They perceived more adequately during the practice and case studies of the EBM course during the surgery clerkship. They stated that they learned the real essence of EBM and became competent in critical appraisal. The course in surgery gave them a chance to practice EBM. This result might indicate that the EBM course had achieved its goals at least in some aspects. Earlier studies also reported the impact of the EBM program on students’ knowledge (8–10, 14), skills (6, 9–14), and attitudes toward learning and applying EBM (10, 11, 13, 14). As with the medical students participating in our study, researchers have also approved that implementing EBM programs during the clinical clerkships may support students in practicing these skills (6, 16).

EBM assignments during both the preclinical years and the surgery clerkship were prepared within a collaborative teamwork. However, half of the students stated that practice of EBM with assignments was moderately sufficient in the preclinical years, while most of the students specified that the course during the surgery clerkship was appropriate for teamwork and contributed to achieving teamwork skills (Table 3). Students thought that the increasing practice of EBM during the clinical years was essential and might provide the integration of knowledge.

## Conclusion

In light of the results of this study, we believe that implementation of EBM during clinical clerkships is critical because the comprehension and application of knowledge is possible when it is integrated with actual patients. Teaching and learning of EBM should certainly be moved from the classroom to clinical settings. However, we also recognize the importance of teaching EBM skills in the early years of the undergraduate medical curriculum in order to prepare students for the clinical years. Even though our students stated that the EBM courses were less utilized during their preclinical years, they indicated that they were acquainted with reading the literature. As a result, students recommended that the EBM courses should be included in the entire curriculum in an integrated manner.
